# Branched-Chain and Aromatic Amino Acids, Type 2 Diabetes, and Cardiometabolic Risk Factors among Puerto Rican Adults

**DOI:** 10.3390/nu16152562

**Published:** 2024-08-04

**Authors:** Sona Rivas-Tumanyan, Lorena S. Pacheco, Danielle E. Haslam, Evangelia Morou-Bermudez, Liming Liang, Katherine L. Tucker, Kaumudi J. Joshipura, Shilpa N. Bhupathiraju

**Affiliations:** 1 Department of Surgical Sciences and the Office of the Assistant Dean for Research, School of Dental Medicine, University of Puerto Rico, San Juan, PR 00936, USA; sona.tumanyan@upr.edu (S.R.-T.);; 2 Department of Nutrition, Harvard T.H. Chan School of Public Health, Boston, MA 02115, USA; 3 Channing Division of Network Medicine, Brigham and Women’s Hospital and Harvard Medical School, 181 Longwood Ave, Boston, MA 02115, USA; 4 Department of Biostatistics, Harvard T.H. Chan School of Public Health, Boston, MA 02115, USA; 5 Department of Biomedical and Nutritional Sciences and Center for Population Health, University of Massachusetts, Lowell, MA 01854, USA; 6 Dean, School of Public Health, Ahmedabad University, Central Campus, Navrangpura, Ahmedabad 380009, Gujarat, India

**Keywords:** branched-chain amino acids, aromatic amino acids, type 2 diabetes, Hispanic, cohort

## Abstract

(1) Background: Branched-chain and aromatic amino acids (BCAAs/AAAs) have been considered as markers of type 2 diabetes (T2D); however, studies on associations between these metabolites and T2D and cardiometabolic traits in Hispanic populations are limited. The aim of this study was to examine the associations between baseline BCAAs (isoleucine, leucine, valine)/AAAs (phenylalanine, tyrosine) and prevalent and incident T2D, as well as baseline and longitudinal (2 year) changes in cardiometabolic traits (measures of glycemia, dyslipidemia, inflammation, and obesity) in two large cohorts of adults of Puerto Rican descent. (2) Methods: We included participants of the Boston Puerto Rican Health Study (BPRHS, *n* = 670) and San Juan Overweight Adult Longitudinal study (SOALS, *n* = 999) with available baseline metabolite and covariate data. T2D diagnosis was defined based on American Diabetes Association criteria. Multivariable logistic (for baseline T2D), Poisson (for incident T2D), and linear (for cardiometabolic traits) regression models were used; cohort-specific results were combined in the meta-analysis and adjusted for multiple comparisons. (3) Results: Higher baseline BCAAs were associated with higher odds of prevalent T2D (OR_1SD BCAA score_ = 1.46, 95% CI: 1.34–1.59, *p* < 0.0001) and higher risk of incident T2D (IRR_1SD BCAA score_ = 1.24, 95% CI: 1.13–1.37, *p* < 0.0001). In multivariable longitudinal analysis, higher leucine and valine concentrations were associated with 2-year increase in insulin (beta _1SD leucine_ = 0.37 mcU/mL, 95% CI: 0.11–0.63, *p* < 0.05; beta _1SD valine_ = 0.43 mcU/mL, 95% CI: 0.17–0.68, *p* < 0.01). Tyrosine was a significant predictor of incident T2D (IRR = 1.31, 95% CI: 1.09–1.58, *p* < 0.05), as well as 2 year increases in HOMA-IR (beta _1SD tyrosine_ = 0.13, 95% CI: 0.04–0.22, *p* < 0.05) and insulin concentrations (beta _1SD tyrosine_ = 0.37 mcU/mL, 95% CI: 0.12–0.61, *p* < 0.05). (4) Conclusions: Our results confirmed the associations between BCAAs and prevalent and incident T2D, as well as concurrent measures of glycemia, dyslipidemia, and obesity, previously reported in predominantly White and Asian populations. Baseline leucine, valine, and tyrosine were predictors of 2 year increases in insulin, whereas tyrosine was a significant predictor of deteriorating insulin resistance over time. Our study suggests that BCAAs and tyrosine could serve as early markers of future glycemic changes in Puerto Ricans.

## 1. Introduction

Diabetes is a prevalent chronic condition and is a major public health concern worldwide. In the US alone, around 38.1 million adults (14.7%) were estimated to be affected by diabetes in 2021, with approximately 90–95% of them having type 2 diabetes (T2D) [1]. Among adults of Hispanic origin, Puerto Ricans were reported to have the highest prevalence of T2D (13.3%) in 2019–2021 [1], surpassing the prevalence among Blacks, Asians, and non-Hispanic Whites. Studies have also shown that the prevalence of T2D risk factors varies by racial and ethnic group [2]. 

Branched-chain amino acids (BCAAs: isoleucine, leucine, valine) are essential amino acids, commonly present in protein-rich foods (e.g., red meat, poultry, eggs, fish, and legumes). The aromatic amino acid group (AAA) includes phenylalanine, tyrosine, and tryptophan, which are also frequently sourced from animal- and plant-based proteins. Increases in fasting circulating BCAAs and phenylalanine and tyrosine have been previously linked to T2D risk [3,4,5,6]. Metabolomic studies have explored BCAA and aromatic amino acids as markers of T2D; however, most were conducted in populations of European or Asian ancestry [7]. A recent meta-analysis of prospective cohort studies confirms positive associations between all individual BCAAs, phenylalanine, tyrosine, and T2D risk [6], with summary relative risk estimates ranging from 1.30 (95% CI: 1.16; 1.45) for phenylalanine to 1.54 (95% CI: 1.36; 1.74) for isoleucine. To date, few studies on metabolites (and particularly BCAAs and AAAs) and incident diabetes in Hispanic populations have been conducted [8,9]. The number of prospective studies focusing on potential underlying mechanisms linking these metabolites and T2D among Hispanics is also limited. Only a few [8,10,11] have examined associations between these metabolites and prospective changes in select cardiometabolic traits, focusing on glycemic changes [8], insulin resistance [10], and anthropometric measures [11]. 

The goal of this study was to explore associations between BCAAs, AAAs, and T2D in two prospective cohorts of Puerto Rican adults, as well as to explore the potential mechanisms underlying these associations through analysis of associations with cardiometabolic risk factors. 

## 2. Materials and Methods

### 2.1. Study Population

The Boston Puerto Rican Health Study (BPRHS) is an ongoing prospective cohort study of Puerto Rican adults recruited from communities in the Greater Boston Area. The details of data collection and recruitment methods of the BPRHS have been published previously [12]. Data were collected by bilingual interviewers during home visits at baseline, 2 years, 3 years, and around 6 years. The primary goal of the original study was to explore the association between psychosocial stress and presence and development of allostatic load and health outcomes among Puerto Ricans. 

The San Juan Overweight Adult Longitudinal Study (SOALS) is a three-year prospective cohort of adult Puerto Ricans residing in San Juan metropolitan area, who were overweight or obese, and free from previous diagnosis of T2D at baseline. The primary goal of this study was to assess the bi-directional association between periodontitis and T2D. SOALS cohort methods have previously been published [13]. 

For the current analyses, we included participants with available plasma BCAA and AAA and covariate data at baseline (*n* = 670 in BPRHS and 999 in SOALS).

### 2.2. T2D Assessment

Participants were categorized as having T2D at baseline or follow-up using the American Diabetes Association (ADA) criteria: having fasting glucose ≥ 126 mg/dL, 2-h post-load glucose ≥ 200 mg/dL (available in SOALS only), or HbA1c ≥ 6.5% [14]. In addition to those criteria, participants were classified as having T2D if they reported a diagnosis by a physician (SOALS) or were taking blood-sugar-lowering medications (BPRHS). 

### 2.3. Metabolomic Profiling

In the BPRHS, fasting blood samples were collected during the home visits, centrifuged, transported to the laboratory on dry ice, processed, and stored at −70 °C. In SOALS, the fasting blood samples were drawn in a laboratory setting, processed, and stored at −80 °C. Blinded baseline plasma specimens from both cohorts were assayed by Metabolon Inc. (Durham, NC, USA) [15]. Raw data were extracted, and peaks were identified and quantified as previously described [16], using area-under-the-curve. Metabolites were identified using a reference library of more than 4500 purified standards for retention time/index, mass-to-charge ratio, and chromatographic data. Data were normalized across all samples and validated by Metabolon, Inc. A mix of pooled matrix sample and a cocktail of quality control standards were included for quality assurance. Samples were randomized across the platform run with QC samples evenly spaced among injections. Undetectable metabolite values were imputed at a value equal to half the minimum of each measured metabolite. Inverse normal transformation was used for all metabolite data.

### 2.4. Cardiometabolic Markers

#### 2.4.1. Measures of Glycemia

In both cohorts, participants were requested to fast for 10 h prior to the study visit [12,13,17]. In SOALS, plasma glucose and insulin concentrations were determined at fasting and 30, 60, and 120 min after glucose load. Glucose levels were determined using a Vitros System 250 instrument (intra-assay coefficient of variation (CV) = 1.21%, inter-assay CV = 3.06%). Plasma insulin concentrations were determined by an immuno-enzymometric assay using a TOSOH analyzer (intra-assay CV = 1.49%; inter-assay CV = 4.42%) [18]. In BPRHS, plasma glucose was measured using an enzymatic, kinetic reaction (OSCR6121; Olympus America, Melville, NY, USA), with intra- and inter-assay CVs of 2% and 3.4%, respectively. Serum insulin was measured using the Immulite 1000 Insulin Kit (LKIN1) on the Immulite 1000 (Siemens Medical Solutions Diagnostics, Los Angeles, CA, USA) [12]. The homeostatic model assessment of insulin resistance (HOMA-IR) was calculated as [fasting glucose (mg/dL) × fasting insulin (mg/dL)]/405] in both cohorts [19]. 

In SOALS, HbA1c was measured with an assay by a latex immunoagglutination inhibition method using the DCA 2000+ Analyzer (Siemens Healthcare Diagnostics, Tarrytown, NY, USA) [18]. In BPRHS, a whole blood hemolysate was analyzed by latex-enhanced immunoturbimetric to determine the HbA1c, and by colorimetric, endpoint for hemoglobin, on the Cobas FARA using the Roche Unimate HbA1c kit (Roche Diagnostics, Indianapolis, Indiana) [12]. 

#### 2.4.2. Measures of Dyslipidemia and Inflammation

In SOALS, triglyceride and high-density lipoprotein cholesterol (HDL-C) concentrations were determined with an enzymatic assay by Roche Diagnostics (Indianapolis, IN, USA) [18]. In BPRHS, HDL-C and triglycerides were analyzed using EDTA plasma with the enzymatic endpoint reaction on the Olympus AU400e with Olympus Reagents (Olympus America Inc., Melville, NY, USA) [12]. Low-density lipoprotein cholesterol (LDL-C) was determined using the Friedewald equation [20]. High-sensitivity C-reactive protein (CRP) was measured with the high sensitive latex turbidimetric method by Beckman Coulter AU5421 K-assay (Beckman Coulter, Inc., Brea, CA, USA) in SOALS [18], using the Immulite 1000 High Sensitive CRP Kit (LKCRP1 on the Immulite 1000 (Siemens Medical Solutions Diagnostics, Los Angeles, CA, USA) in BPRHS [12].

#### 2.4.3. Body Composition Measures

Participants’ height, weight, and waist circumference were measured 2–3 times and later averaged [12,18]. Body mass index (BMI) was calculated using weight (kg), divided by squared height (m).

### 2.5. Assessment of Covariates

Data on covariates were collected during in-person interviews and physical assessments. Information was collected on age, sex, education, smoking status (never, past, current), and alcohol use (grams per week), and physical activity was summarized in terms of metabolic equivalents of task (MET) per week in SOALS and a physical activity score in BPRHS [21]. In SOALS, blood pressure was taken three times after 1–2 min intervals [22] and later averaged. In BPRHS, blood pressure was measured in duplicate, at three time points during the interview, and the second and third readings were averaged. Acculturation was measured in BPRHS by a set of questions on language use in everyday activities. The Perceived Stress Scale (PSS) instrument [23] was used to calculate the total PSS score in BPRHS, with higher values representing higher levels of perceived stress. The American Heart Association (AHA) diet score [24] was calculated for BPRHS participants, using data from validated food frequency questionnaires [25].

### 2.6. Statistical Analysis 

In BPRHS, 736 participants had information on circulating levels of BCAA and AAA metabolites at baseline. Participants were sequentially excluded if they had missing data on diabetes (*n* = 26), or other covariates, including smoking (*n* = 1), BMI (*n* = 6), income (*n* = 30), PSS score (*n* = 1), waist circumference (*n* = 1), and alcohol intake (*n* = 4), resulting in the sample size of 670 for baseline cross-sectional analysis. The analysis on incident diabetes was limited to 316 participants who were free from diabetes at baseline; 43 developed T2D during the 5 years of follow-up. 

In SOALS, 1191 participants completed the baseline and follow-up evaluation and had information on baseline BCAA and AAA metabolites. We excluded those who were missing data on confounders: smoking (*n* = 1), income (*n* = 4), alcohol (*n* = 7), waist circumference (*n* = 1), or physical activity (*n* = 1). Thus, analyses on cross-sectional associations between the metabolites and prevalent diabetes, as well as cardiometabolic risk factors, were limited to 999 participants. For the analysis on incident diabetes, we further excluded SOALS participants who satisfied the definition of diabetes during the baseline evaluation (*n* = 75), resulting in the sample size of 924 participants; 68 developed T2D during 3 years of follow-up. 

Combined BCAA (isoleucine, leucine, valine) and BCAA_AAA (isoleucine, leucine, valine, phenylalanine, tyrosine) scores were calculated as a weighted sum of the corresponding normalized metabolite levels, weighted by multivariable-adjusted logistic regression coefficients for the association between individual metabolites and T2D at baseline [26]. 

To explore associations between metabolites (exposures) and baseline and incident diabetes outcomes, we considered the continuous normalized levels of each metabolite, as well as their tertiles, defined based on the distribution among those free from diabetes at baseline. We further explored associations between baseline metabolites and baseline measures of glycemia (HOMA-IR, fasting insulin, glucose and hemoglobin A1c), dyslipidemia (HDL-C, LDL-C, triglycerides), inflammation (CRP), and anthropometric measures (waist circumference, BMI, and weight) using linear regression models. In addition, we examined associations with 2-year changes in cardiometabolic risk factors, adjusting for baseline measures of the corresponding risk factors. Influential outliers, identified as observations with the Cook’s Distance [27] value exceeding 0.5, were excluded from the corresponding analysis. 

Regression analysis was conducted using SAS statistical software version 9.4 (SAS Institute, Cary, NC, USA). Estimates from two cohorts were combined using an inverse-variance weighted fixed-effects meta-analysis using the *meta* package [28,29] within the R statistical software (V.4.1.3, R Core Team, Vienna, Austria). *p*-values were adjusted for multiple testing using the Benjamini–Hochberg False Discovery Rate (FDR) correction method [30]; statistical significance was established at α = 0.05 level. 

## 3. Results

Compared to SOALS, BPRHS participants were slightly older at baseline, more likely to have less than 9th grade schooling, to be past or current smokers, and to use medications for hypertension (Table 1).

### 3.1. BCAAs, AAAs, and Prevalent and Incident T2D

**Figure 1:**
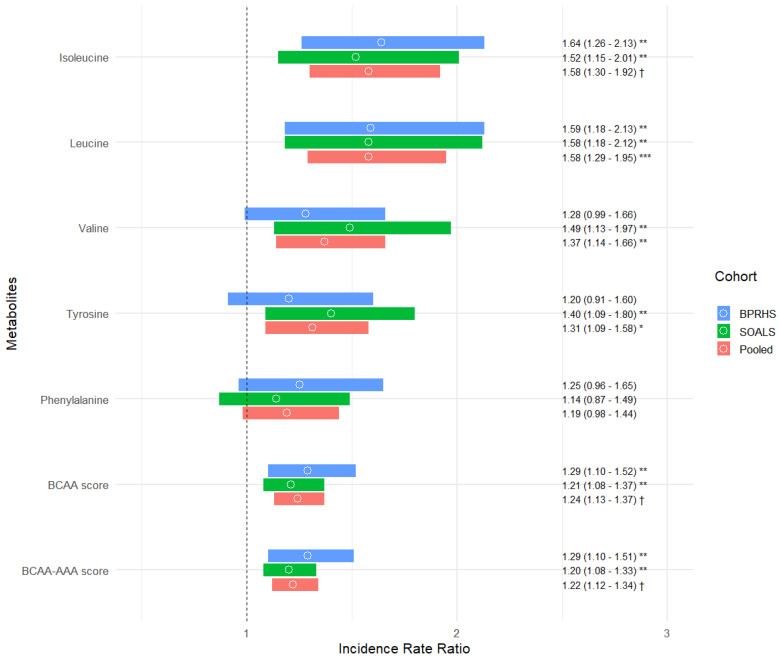
Associations between branched-chain amino acids and aromatic amino acids, and incident type 2 diabetes in the Boston Puerto Rican Health Study (BPRHS, *n* = 316) and San Juan Overweight Adult Longitudinal Study (SOALS, *n* = 924). FDR-adjusted *p*-value: * *p* < 0.05; ** *p* < 0.01; *** *p* < 0.001; † *p* < 0.0001. Incidence rate ratio estimates were obtained from Poisson regression models, using the log-transformed follow-up time as the offset variable. Branched-chain amino acid (BCAA) score is a combined metabolite score including leucine, isoleucine, and valine, weighted according to the strength of the association of each metabolite with T2D. Branched-chain amino acids and aromatic amino acids (BCAA-AAA) score is a combined metabolite score including leucine, isoleucine, valine, phenylalanine, and tyrosine, weighted according to the strength of the association of each metabolite with T2D. BPRHS multivariable model adjusted for age, sex, education, income, smoking, alcohol consumption, physical activity score, acculturation, perceived stress score, multivitamin use, anti-hypertensive and lipid-lowering medication use, American Heart Association diet score, 2-year change in waist circumference, and body mass index. SOALS multivariable model adjusted for age, sex, education, income, smoking, alcohol consumption, physical activity, anti-hypertensive and lipid-lowering medication use, 2-year change in waist circumference, and body mass index.

In cross-sectional analysis, participants in the highest tertile of the three BCAA metabolites had a nearly four-times higher odds of diabetes (Table S1) compared to those in the lowest tertile. In the combined meta-analysis, 1 SD increase in leucine was associated with an OR of 2.07 for baseline diabetes (95% CI: 1.75; 2.45). Baseline T2D OR estimates for 1 SD increase in isoleucine, valine, the combined BCAA score, and the BCAA-AAA score were similar, ranging from 1.42 (for the BCAA-AAA score) to 1.94 (for isoleucine). In the longitudinal analysis (Figure 1), all metabolites were significantly associated with increased risk of incident T2D. The strongest associations were found for 1 SD increase in baseline leucine (pooled IRR = 1.58, 95% CI: 1.29; 1.95, FDR-adjusted *p* < 0.001) and isoleucine (pooled IRR = 1.58, 95% CI: 1.30; 1.92, FDR-adjusted *p* < 0.0001), followed by valine (pooled IRR = 1.37, 95% CI: 1.14; 1.66, FDR-adjusted *p* < 0.01) and tyrosine (pooled IRR = 1.31, 95% CI: 1.09; 1.58, FDR-adjusted *p* < 0.05).

### 3.2. BCAAs, AAAs, and Baseline Cardiometabolic Risk Factors

All BCAA metabolite levels were strongly and positively associated with baseline measures of glycemia, such as fasting HOMA-IR, insulin, glucose, and HbA1c (Table S2). Phenylalanine and tyrosine were positively associated with HOMA-IR and insulin; however, there were no associations between these aromatic amino acids and glucose and glycosylated hemoglobin. 

BCAAs and AAAs were negatively associated with baseline HDL-C (Table S2). Triglycerides, on the other hand, were positively associated with all metabolites, with beta coefficient estimates for 1 SD increase in BCAAs associated with 18–20 mg/dL increase in triglyceride concentration. The associations between BCAAs, aromatic amino acids, and baseline LDL-C concentration were not consistent, ranging from no association (isoleucine, tyrosine) to a positive association (valine). There were no meaningful associations between the tested metabolites and CRP, except for phenylalanine showing a weak positive association (Table S2).

Valine, phenylalanine, and tyrosine showed a significant positive association with baseline waist circumference, BMI, and weight. In pooled analysis, there were no associations between isoleucine, leucine, and the anthropometric measures included in this analysis (Table S2). 

### 3.3. BCAAs, AAAs, and Longitudinal Changes in Cardiometabolic Risk Factors

In the longitudinal analysis, all three BCAAs were associated with an increase in fasting insulin and glucose concentrations over time, but not with changes in insulin resistance (as measured by ΔHOMA-IR) or HbA1c (Table 2). Furthermore, after FDR adjustment, leucine and valine remained significantly associated with increases in insulin over time (pooled beta coefficient for leucine: 0.37 mcU/mL, 95% CI: 0.11–0.63; for valine: 0.43 mcU/mL, 95% CI: 0.17–0.68). Tyrosine showed a positive association with both insulin and HOMA-IR changes after FDR adjustment. 

Leucine and valine appeared to be negatively associated with changes in HDL-C; however, the associations were no longer statistically significant after FDR adjustment. There were no associations between BCAAs, AAAs, and longitudinal changes in triglycerides or CRP (Table 2).

Phenylalanine and tyrosine were associated with increased waist circumference over time, and isoleucine appeared to be negatively associated with increased BMI, but only prior to FDR adjustment. There were no associations between the metabolites and changes in anthropometric measurements after FDR adjustments.

## 4. Discussion

This study was designed to take a deeper look at the relationship between BCAAs, AAAs, and cardiometabolic risk through analysis of cross-sectional and longitudinal associations with clinical outcomes (using the ADA definition of T2D), as well as prognostically relevant risk factors, such as markers of glucose and lipid metabolism, inflammation, and obesity. In the analysis of two prospective cohorts among individuals of Puerto Rican descent, we confirmed strong cross-sectional and longitudinal associations between the BCAA and AAA metabolites and T2D, as well as cross-sectional positive associations with baseline markers of glucose metabolism, with further suggestion of a potential association of these metabolites with increases in fasting insulin and glucose over time. 

Isoleucine, leucine, and valine (and their derivatives, to some extent) have been studied extensively in relation to T2D risk in prospective observational cohorts [3,4,5,6] as well as in studies using Mendelian randomization methods [31,32]. The existing literature is consistent in identifying these metabolites as markers for prevalent, as well as incident, T2D in populations of European and Asian [6], and to a lesser extent, Hispanic descent [4,8,9,33]. A recent meta-analysis [6] confirmed significant associations between individual BCAAs, AAAs, and prevalent and incident T2D, and it reported a summary of the relative risk estimates that were very similar to our findings: 1.54 (95% CI: 1.36; 1.74) for isoleucine, 1.40 (95% CI: 1.29; 1.52) for leucine, 1.40 (95% CI: 1.25; 1.57) for valine, 1.30 (95% CI: 1.16; 1.45) for phenylalanine, and 1.35 (95% CI: 1.22; 1.49) for tyrosine. 

While T2D disproportionally affects Hispanic populations, this report is one of the few focusing primarily on Puerto Rican adults. Our previous studies have focused on network-derived metabolic clusters in relation to baseline diabetes status [16], as well as incident diabetes [9], in these two cohorts. In these studies, the combined cluster of 13 metabolites of BCAA and AAA metabolism was associated with progression to incident diabetes (pooled IRR = 1.87, 95% CI: 1.28; 2.73). A recent analysis on serum metabolites and glycemic changes in the Hispanic Community Health study/Study of Latinos (SOL), which includes several Hispanic subgroups (Dominican, Central and South American, Cuban, Mexican, and Puerto Rican), reported similar results on the effect of these metabolites on incident diabetes risk, with the adjusted RR for the BCAA/AAA/g-glutamyl amino acid (GGAA) cluster estimated at 1.57 (95% CI: 1.26–1.95), with some variation across Hispanic background subgroups [8]. 

The current literature does not provide a consensus regarding the biological mechanisms linking BCAAs to cardiometabolic risk factors, nor the direction of these associations. Increased leucine has been hypothesized to lead to insulin resistance through activation of the Target of Rapamycin (TOR) complex 1 pathway, with modulation of beta cell proliferation and insulin secretion [34] and disruption of insulin signaling in skeletal muscle [35]. In addition, earlier undetected changes in insulin resistance have been postulated to affect BCAA levels [36], possibly through hyperglycemia-led suppression of adipose tissue expression of genes involved in branched-chain amino acid oxidation, which in turn can contribute to increased levels of BCAA [37]. Notably, the gut microbiome, in particular Prevotella and Bacteroides species, have been proposed as mediators between the biosynthesis of BCAAs and insulin resistance [38]. Recent studies suggest that changes in gut microbiota in obesity, in particular the reduction of species of Bacteroides involved in fermentation of AAAs and abundance of other Bacteroidetes that participate biosynthesis of BCAAs, may play a pivotal role in production of BCAAs and AAAs, as well as in subsequent insulin resistance [39]. Regardless of the pathways explaining these associations, BCAAs are viewed as an early marker of pre-clinical metabolic changes, as well as being the underlying mechanisms connecting the gut microbiome with obesity and insulin resistance [39].

Our results from the cross-sectional analysis on BCAAs and markers of glucose metabolism are consistent with the body of literature from other populations, demonstrating strong positive associations. Our longitudinal analysis, on the other hand, suggests that BCAA contribute to 2 year increases in fasting insulin (FDR-adjusted *p* < 0.05 for leucine, valine, tyrosine, BCAA score, and BCAA-AAA score) and glucose (*p* < 0.05 only prior to FDR adjustment), but not in HOMA-IR, the combined measure of insulin resistance. Changes in HOMA-IR, however, were associated with baseline tyrosine levels (beta = 0.13, 95% CI: 0.04; 0.22, FDR-adjusted *p* < 0.05) and the combined BCAA-AAA metabolite score (beta = 0.04, 95% CI: 0.01; 0.08, *p* < 0.05 prior to FDR adjustment). In a recent study by Chai et al. [8], one of the few studies conducted in Hispanic populations, the combined module of 38 metabolites of BCAA, AAA, and GGAA metabolism was associated with changes in cardiometabolic risk factors, such as HOMA-IR (beta = 3.35, 95% CI: 0.32; 6.38, *p* = 0.03) and fasting glucose (beta = 1.79, 95% CI: 0.49; 3.09, *p* < 0.01), but not insulin (beta = 3.14, 95% CI: −0.44; 6.72, *p* = 0.09). This study, however, did not report on estimates for individual BCAAs and AAAs in relation to cardiometabolic traits. In addition, Chai et al. [8] focused on six-year changes in risk factors (vs. two-year changes in BPRHS/SOALS), which could also potentially explain the differences in our findings. 

Like previous cross-sectional reports [40], we found significant associations between assessed BCAAs, AAAs, and lower HDL-C, as well as higher triglycerides. C-reactive protein, a marker of inflammation, was associated with phenylalanine only.

Previous studies have demonstrated higher BCAAs among obese vs. healthy-weight individuals in cross-sectional settings [41,42], with a linear trend reported between BMI categories and BCAA [43]. In a five-year follow-up study among Mexican American healthy women, Zhao et al. [11] reported significant associations between baseline leucine level and weight gain (defined as an increase in BMI by at least one category) in both training (HR for weight gain = 2.23, 95% CI: 1.31, 3.80, *p* < 0.0001) and testing cohorts (HR = 1.54, 95% CI: 1.09, 5.23, *p* = 0.025). In our study, cross-sectional associations with anthropometric measures were strong for valine, phenylalanine, and tyrosine, as well as the combined BCAA and BCAA-AAA scores. Nevertheless, longitudinal associations were attenuated after FDR adjustment, suggesting that overweight/obesity is more likely to contribute to increased BCAA levels, rather than the other way around. Recent studies suggest that changes in gut microbiota in obesity, in particular the reduction of Bacteroides involved in fermentation of AAAs and abundance of Bacteroidetes that participate biosynthesis of BCAAs, may play a pivotal role in production of BCAAs and AAAs, as well as in subsequent insulin resistance [39].

Our study has several strengths. The current analysis is one of the few to evaluate several potential mechanisms to explain the predictive role BCAAs and AAAs in development of T2D, through evaluation of cross-sectional and longitudinal associations with measures of glucose metabolism, dyslipidemia, inflammation, and obesity. We assessed the targeted metabolites individually, as well as in combination scores, which allows for testing for differential effects of these metabolite pathways. Availability of data on potential confounders allowed for the adjustment for multiple risk factors in our regression analysis. Our analysis included two cohorts of adults of Puerto Rican descent, which limits potential variability of effect estimates by racial/ethnic background.

## 5. Conclusions

In this analysis of two cohorts of Puerto Ricans, we found strong associations between baseline BCAA and AAA metabolites and T2D at baseline and follow-up, confirming their potential prognostic role in diabetes development among Puerto Ricans. Our longitudinal analysis of cardiometabolic risk factors showed that baseline leucine, valine, and tyrosine were predictors of two-year change increases in insulin concentration, whereas tyrosine was also a predictor of deteriorating insulin resistance over time (HOMA-IR). Our study suggests that BCAAs (particularly leucine and valine) and tyrosine could serve as early markers of future glycemic changes in Puerto Ricans.

## Figures and Tables

**Table 1:** Characteristics of the Boston Puerto Rican Health Study (BPRHS) and San Juan Overweight Adult Longitudinal Study (SOALS) adults at baseline.

	BPRHS (*n* = 670)	SOALS (*n* = 999)
	Mean (±SD) or *n* (%)	Mean (±SD) or *n* (%)
Age, years	57.2 (±7.41)	50.7 (±6.77)
Female	502 (74.9)	729 (73.0)
Total income, USD/dollars	18,003 (±18,221)	-
<20,000	-	543 (54.4)
20,000–49,999	-	338 (33.8)
≥50,000	-	118 (11.8)
Education		
No schooling–7th to 8th grade	336 (50.2)	113 (11.3)
9th–12th grade	237 (35.4)	439 (43.9)
Some college or more	97 (14.5)	447 (44.7)
Smoking status		
Never	315 (47.0)	639 (64.0)
Past	204 (30.5)	179 (17.9)
Current	151 (22.5)	181 (18.1)
Alcohol consumption		
Never/abstainer	203 (30.3)	442 (44.2)
Past	197 (29.4)	113 (11.3)
Current	270 (40.3)	444 (44.4)
Multivitamin supplement use, yes	134 (20.0)	-
Statin or lipid-lowering medication use, yes	295 (44.0)	85 (8.51)
Hypertension medication use, yes	376 (56.1)	267 (26.7)
BMI, kg/m^2^	32.2 (±6.67)	33.3 (±6.17)
Waist circumference, cm	102 (±14.9)	106 (±14.0)
LDL cholesterol, mg/dL	108 (±34.4)	123 (±32.7)
HDL cholesterol, mg/dL	45.2 (±12.4)	48.1 (±13.1)
Triglycerides, mg/dL	162 (±112)	149 (±83.7)
Glucose, mg/dL	120 (±50.2)	95.8 (±20.2)
Hemoglobin A1c, %	7.00 (±1.78)	5.80 (±0.62)
HOMA-IR	6.06 (±9.99)	2.62 (±1.83)
Insulin, mcU/mL	18.8 (±26.2)	10.8 (±6.83)
C-reactive protein, mg/L	6.36 (±8.83)	5.92 (±6.32)
Type 2 diabetes, yes	354 (52.84)	75 (7.51)
Systolic blood pressure, mmHg	136 (±18.8)	129 (±17.1)
Diastolic blood pressure, mmHg	81.5 (±10.7)	80.9 (±9.67)
Physical activity	31.4 (±4.40)	22.0 (±39.7)
Alcohol, g/d	4.05 (±15.4)	2.36 (±5.82)
AHA diet score	8.70 (±2.04)	-
Psychosocial stress score	23.4 (±9.67)	-
Cultural acculturation score	22.6 (±21.2)	-

LDL, low-density lipoprotein; HDL, high-density lipoprotein; AHA, American Heart Association.

**Table 2:** Pooled beta coefficients (95% confidence intervals) for longitudinal changes in cardiometabolic trait measures, according to 1 SD increase in baseline branched-chain and aromatic amino acids in the Boston Puerto Rican Health Study (BPRHS) and San Juan Overweight Longitudinal Study (SOALS).

**2 YEAR CHANGES IN GLYCEMIC MEASURES**				
**Metabolite**	**Δ HOMA-IR**	**Δ Insulin, mcU/mL**	**Δ Glucose, mg/dL**	**Δ HbA1c,** **%**
Isoleucine	0.07 (−0.03; 0.17)	**0.28** **(0.03; 0.54)**	**0.89** **(0.22; 1.56)**	0.02 (−0.001; 0.04)
Leucine	0.09 (−0.008; 0.19)	**0.37** (0.11; 0.63) *	**0.78** **(0.09; 1.46)**	0.02 (−0.004; 0.04)
Valine	0.09 (−0.003; 0.19)	**0.43** (0.17; 0.68) **	**0.67** **(0.02; 1.33)**	0.01 (−0.01; 0.03)
Phenylalanine	0.04 (−0.05; 0.14)	0.24 (−0.01; 0.48)	0.01 (−0.63; 0.65)	0.01 (−0.01; 0.03)
Tyrosine	**0.13** (0.04; 0.22) *	**0.37** (0.12; 0.61) *	0.36 (−0.28; 1.00)	0.01 (−0.01; 0.03)
BCAA score ^a^	0.04 (−0.001; 0.08)	**0.18** (0.06; 0.30) *	**0.36** **(0.06; 0.67)**	0.01 (−0.003; 0.02)
BCAA-AAA score ^b^	**0.04** **(0.01; 0.08)**	**0.19** (0.08; 0.30) **	**0.30** **(0.03; 0.58)**	0.01 (−0.003; 0.01)
**2 YEAR CHANGES IN DYSLIPIDEMIA AND INFLAMMATION MEASURES**				
**Metabolite**	**Δ HDL−C,** **mg/dL**	**Δ LDL−C,** **mg/dL**	**Δ Triglycerides,** **mg/dL**	**Δ CRP,** **mg/L**
Isoleucine	−0.19 (−0.41; 0.03)	−0.03 (−0.78; 0.73)	1.62 (−0.22; 3.46)	−0.05 (−0.24; 0.14)
Leucine	**−0.28** **(−0.51; −0.05)**	−0.13 (−0.90; 0.65)	1.62 (−0.27; 3.51)	−0.08 (−0.28; 0.11)
Valine	**−0.24** **(−0.46; −0.02)**	0.02 (−0.74; 0.77)	1.31 (−0.5; 3.13)	0.01 (−0.17; 0.20)
Phenylalanine	−0.21 (−0.42; 0.01)	−0.24 (−0.97; 0.50)	0.33 (−1.43; 2.09)	−0.11 (−0.30; 0.08)
Tyrosine	0.04 (−0.18; 0.25)	−0.26 (−0.99; 0.48)	−0.18 (−1.95; 1.58)	−0.07 (−0.25; 0.11)
BCAA score ^a^	**−0.15** **(−0.27; −0.03)**	0.01 (−0.38; 0.40)	0.90 (−0.03; 1.83)	−0.01 (−0.11; 0.08)
BCAA-AAA score ^b^	**−0.14** **(−0.25; −0.03)**	−0.02 (−0.38; 0.34)	0.77 (−0.09; 1.63)	−0.01 (−0.10; 0.07)
**2 YEAR CHANGES IN ANTHROPOMETRIC MEASURES**				
**Metabolite**	**Δ Waist Circumference, cm**	**Δ BMI**	**Δ Weight,** **kg**	
Isoleucine	0.10 (−0.20; 0.39)	**−0.09** **(−0.17; −0.01)**	−0.15 (−0.38; 0.09)	
Leucine	0.16 (−0.14; 0.47)	−0.08 (−0.16; 0.005)	−0.07 (−0.31; 0.18)	
Valine	0.15 (−0.14; 0.44)	−0.03 (−0.11; 0.05)	0.05 (−0.18; 0.29)	
Phenylalanine	**0.37** **(0.08; 0.65)**	−0.06 (−0.14; 0.02)	−0.04 (−0.27; 0.19)	
Tyrosine	**0.30** **(0.02; 0.58)**	−0.02 (−0.10; 0.06)	−0.12 (−0.35; 0.12)	
BCAA score ^a^	0.08 (−0.06; 0.22)	−0.04 (−0.08; 0.0002)	−0.04 (−0.18; 0.09)	
BCAA-AAA score ^b^	0.09 (−0.04; 0.22)	**−0.04** **(−0.08; −0.001)**	−0.05 (−0.18; 0.08)	

FDR-adjusted *p*-value: * *p* < 0.05; ** *p* < 0.01. Estimates were highlighted in **bold** if the *p*-value was <0.05 before the FDR adjustment. Values represent unit change in outcome per SD increase in metabolite. ^a^ Branched-chain amino acid score is a combined metabolite score including leucine, isoleucine, and valine, weighted according to the strength of the association of each metabolite with T2D. ^b^ Branched-chain amino acid and aromatic amino acid score is a combined metabolite score including leucine, isoleucine, valine, phenylalanine, and tyrosine, weighted according to the strength of the association of each metabolite with T2D, weighted according to the strength of the association of each metabolite with T2D. BPRHS multivariable models adjusted for baseline levels of the corresponding measure and participant’s age, sex, education, income, smoking, physical activity, alcohol consumption, acculturation, perceived-stress score, multivitamin use, antihypertensive and lipid-lowering medication use, American Heart Association diet score, baseline body mass index, and baseline diabetes status. SOALS multivariable models adjusted for baseline levels of the corresponding measure and participant’s age, sex, education, income, smoking, physical activity, alcohol consumption, anti-hypertensive and lipid-lowering medication use, baseline body mass index, and baseline diabetes status. Additionally for glycemic, dyslipidemia, and inflammation measures in both cohorts, the models included 2-year changes in waist circumference. Models for changes in anthropometric measures additionally adjusted for baseline waist circumference. Abbreviations: HbA1c, hemoglobin A1c. HDL-C, high-density lipoprotein cholesterol. LDL-C, low-density lipoprotein cholesterol. CRP, c-reactive protein. BMI, body mass index.

## Data Availability

Data are available upon reasonable request, following approval of the analysis plan from the study investigators. For more information on requesting data from the BPRHS (https://www.uml.edu/Research/UML-CPH/Research/bprhs/, accessed on 20 June 2024) and SOALS (http://soals.rcm.upr.edu/, accessed on 20 June 2024), please refer to their respective websites.

## Supplementary Materials

The following supporting information can be downloaded at https://www.mdpi.com/article/10.3390/nu16152562/s1. Table S1. Pooled odds ratios (95% confidence intervals) for prevalent type 2 diabetes by branched-chain amino acids and aromatic amino acids in the Boston Puerto Rican Health Study (BPRHS, *n* = 670) and San Juan Overweight Adult Longitudinal Study (SOALS, *n* = 999). Table S2. Pooled beta coefficients (95% confidence intervals) for cardiometabolic traits at baseline, according to 1 SD increase in branched-chain and aromatic amino acids in the Boston Puerto Rican Health Study (BPRHS) and San Juan Overweight Adult Longitudinal Study (SOALS).

## Abbreviations

AAA, aromatic amino acid; ADA, American Diabetes Association; AHA, American Heart Association; BCAA, branched-chain amino acid; BMI, body mass index; BPRHS, Boston Puerto Rican Health Study; CI, confidence interval; CRP, C-reactive protein; CV, coefficient of variation; FDR, false discovery rate; GGAA, g-glutamyl amino acid; HbA1c, glycated hemoglobin %; HDL-C, high-density lipoprotein cholesterol; HOMA-IR, homeostatic model assessment for insulin resistance; HR, hazard ratio; IRR, incidence rate ratio; LDL-C, low-density lipoprotein cholesterol; MET, metabolic equivalent of task; OR, odds ratio; PSS, Perceived Stress Scale; SD, standard deviation; SOALS, San Juan Overweight Adult Longitudinal Study; T2D, type 2 diabetes.
