# Supplementary material for: Branched-Chain and Aromatic Amino Acids, Type 2 Diabetes, and Cardiometabolic Risk Factors among Puerto Rican Adults
Source: Nutrients. 2024 Aug 4;16(15):2562. doi: 10.3390/nu16152562 (PMC11313859; doi:10.3390/nu16152562)
Supplement: Supplementary file 1 [file nutrients-16-02562-s001.zip › nutrients-3092586-supplementary.pdf]

Supplemental Tables

Table S1. Pooled odds ratios (95% confidence intervals) for prevalent type 2 diabetes by branched-chain amino acids and aromatic amino acids in the Boston Puerto Rican Health Study (BPRHS, n=670) and San Juan Overweight Adult Longitudinal Study (SOALS, n=999).

Metabolite	Tertile 2	Tertile 3	Per SD
**Isoleucine**			
BPRHS	**2.38 (1.51, 3.77)*** **	**3.54 (2.22, 5.65)** †	**1.82 (1.50, 2.20)** †
SOALS	1.95 (0.85; 4.43)	**5.07 (2.34; 11.00)** †	**2.28 (1.70, 3.07)** †
Pooled	**2.27 (1.52, 3.39)** †	**3.90 (2.61, 5.81)** †	**1.94 (1.66, 2.28)** †
**Leucine**			
BPRHS	1.42 (0.91, 2.21)	**3.15 (2.01, 4.93)** †	**1.94 (1.59, 2.36)** †
SOALS	1.38 (0.60; 3.17)	**4.97 (2.37; 10.44)** †	**2.44 (1.79, 3.32)** †
Pooled	1.41 (0.95, 2.08)	**3.56 (2.42, 5.22)** †	**2.07 (1.75, 2.45)** †
**Valine**			
BPRHS	1.47 (0.94, 2.32)	**2.94 (1.88, 4.58)** †	**1.71 (1.42, 2.06)** †
SOALS	**3.16 (1.32; 7.53)***	**6.10 (2.64; 14.13)** †	**2.18 (1.64, 2.90)** †
Pooled	1.73 (1.16, 2.59)	**3.45 (2.33, 5.11)** †	**1.84 (1.57, 2.15)** †
**Phenylalanine**			
BPRHS	1.13 (0.75, 1.71)	1.10 (0.73, 1.66)	1.04 (0.88, 1.24)
SOALS	1.86 (0.93; 3.73)	**2.27 (1.13; 4.55)***	1.27 (0.97, 1.66)
Pooled	1.29 (0.90, 1.84)	1.33 (0.93, 1.90)	1.11 (0.96, 1.28)
**Tyrosine**			
BPRHS	0.72 (0.47, 1.09)	0.97 (0.65, 1.44)	1.00 (0.84, 1.18)
SOALS	0.96 (0.46; 1.99)	**2.43 (1.29; 4.59)****	**1.41 (1.09, 1.83)***
Pooled	0.77 (0.54, 1.11)	1.26 (0.90, 1.77)	1.11 (0.96, 1.27)
**BCAA[a] score**			
BPRHS	**1.95 (1.24, 3.07)****	**3.32 (2.10, 5.24)** †	**1.47 (1.31, 1.65)** †
SOALS	**3.13 (1.31, 7.51)***	**6.22 (2.66, 14.52)** †	**1.46 (1.29, 1.65)** †
Pooled	**2.04 (1.36, 3.06)****	**3.93 (2.63, 5.87)** †	**1.46 (1.34, 1.59)** †
**BCAA-AAA[b] score**			
BPRHS	**2.03 (1.28, 3.21)****	**3.47 (2.18, 5.53)** †	**1.45 (1.30, 1.63)** †
SOALS	**2.46 (1.01, 6.03)**	**6.98 (3.00, 16.25)** †	**1.39 (1.24, 1.56)** †
Pooled	**2.23 (1.48, 3.34)****	**3.97 (2.64, 5.97)** †	**1.42 (1.31, 1.54)** †

FDR-adjusted p-value: * p <0.05; ** p <0.01; *** p <0.001; † p <0.0001. Estimates were highlighted in **bold** if the p-value was <0.05 before the FDR adjustment.

Odds ratio estimates were obtained from logistic regression models.
[a] Branched-chain amino acid score is a combined metabolite score including leucine, isoleucine, and valine, weighted according to the strength of the association of each metabolite with T2D.

[b] Branched-chain amino acid and aromatic amino acid score is a combined metabolite score including leucine, isoleucine, valine, phenylalanine, and tyrosine, weighted according to the strength of the association of each metabolite with T2D.

BPRHS multivariable models adjusted for age, sex, smoking status, education, physical activity score, alcohol intake, income, acculturation, perceived-stress score, multivitamin use, and body mass index.

SOALS multivariable models adjusted for age, sex, smoking status, education, physical activity score, alcohol intake, income, and body mass index.

Table S2. Pooled beta coefficients (95% confidence intervals) for cardiometabolic traits at baseline, according to 1 SD increase in branched-chain and aromatic amino acids in the Boston Puerto Rican Health Study (BPRHS) and San Juan Overweight Adult Longitudinal Study (SOALS).

**BASELINE GLYCEMIC MEASURES**				
**Metabolite**	**HOMA-IR**	**Insulin, mcU/mL**	**Glucose, mg/dL**	**HbA1c, %**
**Isoleucine**	**0.46 (0.36; 0.57)†**	**1.76 (1.35; 2.16)†**	**2.38 (1.3; 3.46)*** **	**0.07 (0.03; 0.10)*** **
**Leucine**	**0.42 (0.31; 0.53)†**	**1.51 (1.09; 1.93)†**	**2.66 (1.56; 3.76)†**	**0.08 (0.05; 0.12)†**
**Valine**	**0.47 (0.36; 0.57)†**	**1.69 (1.29; 2.09)†**	**2.67 (1.61; 3.72)†**	**0.09 (0.05; 0.12)†**
**Phenylalanine**	**0.31 (0.21; 0.42)†**	**1.35 (0.96; 1.74)†**	0.14 (-0.90; 1.18)	0.02 (-0.02; 0.05)
**Tyrosine**	**0.46 (0.36; 0.56)†**	**1.84 (1.46; 2.22)†**	0.97 (-0.05; 2.00)	0.02 (-0.01; 0.06)
**BCAA score[a]**	**0.21 (0.16; 0.25)†**	**0.76 (0.59; 0.94)†**	**1.14 (0.68; 1.61)†**	**0.04 (0.02; 0.05)†**
**BCAA-AAA score[b]**	**0.20 (0.16; 0.24)†**	**0.75 (0.6; 0.91)†**	**0.94 (0.53; 1.36)†**	**0.03 (0.02; 0.05)†**
**BASELINE DYSLIPIDEMIA AND INFLAMMATION MEASURES**				
**Metabolite**	**HDL-C, mg/dL**	**LDL-C, mg/dL**	**Triglycerides, mg/dL**	**CRP, mg/L**
**Isoleucine**	**-2.27 (-2.91; -1.64)†**	0.06 (-1.71; 1.83)	**19.86 (15.03; 24.69)†**	0.10 (-0.24; 0.44)
**Leucine**	**-1.90 (-2.55; -1.25)†**	1.37 (-0.43; 3.17)	**19.07 (14.14; 23.99)†**	0.13 (-0.22; 0.47)
**Valine**	**-1.71 (-2.34; -1.08)†**	**2.81 (1.08; 4.55)***	**18.09 (13.34; 22.84)†**	0.13 (-0.20; 0.47)
**Phenylalanine**	**-1.47 (-2.08; -0.85)†**	**-1.52 (-3.21; 0.18)**	**5.27 (0.56; 9.98)**	**0.57 (0.24; 0.89)****
**Tyrosine**	**-1.21 (-1.82; -0.60)†**	-0.97 (-2.65; 0.7)	**5.64 (1.00; 10.28)**	0.27 (-0.05; 0.59)
**BCAA score[a]**	**-1.01 (-1.31; -0.70)†**	0.83 (-0.01; 1.67)	**9.31 (7.11; 11.51)†**	0.07 (-0.09; 0.22)
**BCAA-AAA score[b]**	**-0.96 (-1.24; -0.68)†**	0.56 (-0.20; 1.32)	**8.05 (6.07; 10.03)†**	0.09 (-0.05; 0.23)
**BASELINE ANTHROPOMETRIC MEASURES**				

Metabolite	Waist Circumference, cm	BMI	Weight, kg	
**Isoleucine**	0.64<br>(-0.09; 1.38)	0.18<br>(-0.15; 0.51)	**0.93**<br>**(0.01; 1.86)**	
**Leucine**	0.7<br>(-0.05; 1.45)	0.23<br>(-0.10; 0.57)	**1.20**<br>**(0.26; 2.14)**	
**Valine**	**1.95**<br>**(1.24; 2.66)†**	**0.87**<br>**(0.56; 1.19)†**	**2.69**<br>**(1.8; 3.59)†**	
**Phenylalanine**	**2.23**<br>**(1.54; 2.91)†**	**1.09**<br>**(0.79; 1.4)†**	**3.35**<br>**(2.50; 4.21)†**	
**Tyrosine**	**2.55**<br>**(1.88; 3.22)†**	**1.25**<br>**(0.95; 1.55)†**	**3.72**<br>**(2.88; 4.56)†**	
**BCAA score[a]**	**0.53**<br>**(0.19; 0.88)***	**0.21**<br>**(0.06; 0.37)***	**0.81**<br>**(0.37; 1.25)****	
**BCAA-AAA score[b]**	**0.63**<br>**(0.32; 0.95)*****	**0.28**<br>**(0.14; 0.42)*****	**0.96**<br>**(0.56; 1.36)†**	

FDR-adjusted p-value: * p <0.05; ** p <0.01; *** p <0.001; † p <0.0001. Estimates were highlighted in **bold** if the p-value was <0.05 before the FDR adjustment.

Beta coefficient values represent unit change in outcome over a two-year period per SD increase in metabolites.

HbA1c, hemoglobin A1c.

HDL-C, high-density lipoprotein cholesterol.

LDL-C, low-density lipoprotein cholesterol.

CRP, c-reactive protein.

BMI, body mass index.

[a] Branched-chain amino acid score is a combined metabolite score including leucine, isoleucine, and valine, weighted according to the strength of the association of each metabolite with T2D.

[b] Branched-chain amino acid and aromatic amino acid score is a combined metabolite score including leucine, isoleucine, valine, phenylalanine, and tyrosine, weighted according to the strength of the association of each metabolite with T2D.

All BPRHS multivariable models adjusted for age, sex, smoking status, education, physical activity, alcohol intake, income, acculturation, perceived stress score, multivitamin use, antihypertensive and lipid-lowering medication use, American Heart Association diet score, and baseline diabetes status.

All SOALS multivariable models adjusted for age, sex, smoking status, education, physical activity, alcohol intake, income, anti-hypertensive medication use, use of lipid-lower medications, and baseline diabetes status.

Additionally for glycemic, dyslipidemia, and inflammation measures, models included waist circumference and body mass index.
